# Cytocompatibility and Antibiofilm Activity of Calcium Hydroxide Mixed with *Cyperus articulatus* Essential Oil and Bio-C Temp Bioceramic Intracanal Medicament

**DOI:** 10.3390/antibiotics13070637

**Published:** 2024-07-10

**Authors:** Cláudia Fernandes de Magalhães Silveira, Carlos Eduardo da Silveira Bueno, Angélica Zaninelli Schreiber

**Affiliations:** 1 School of Medical Sciences, State University of Campinas—UNICAMP, Campinas 13083-970, SP, Brazil; zaninele@unicamp.br; 2 Department of Endodontics, Faculdade São Leopoldo Mandic, Instituto de Pesquisas São Leopoldo Mandic, Campinas 13045-755, SP, Brazil; carlosesbueno@terra.com.br

**Keywords:** antimicrobial activity, bioceramics, biofilm, calcium hydroxide, *Cyperus articulatus*, endodontics, *Enterococcus faecalis*, toxicity assessment

## Abstract

Calcium hydroxide represents the most commonly used intracanal dressing between sessions; however, it may not be effective against all types of microorganisms. Several compounds of plant origin have attracted increasing attention from researchers in recent years. The objective of this study was to evaluate the cytocompatibility and antimicrobial activity of calcium hydroxide associated with the essential oil of *Cyperus articulatus* and the new bioceramic intracanal medicament Bio-C Temp^®^. Five experimental groups were designed: group Ca–*C. articulatus* essential oil; group CHPG-calcium hydroxide associated with propylene glycol; group CHCa-essential oil of *C. articulatus* associated with calcium hydroxide; and group U-UltraCal^®^ XS; group BCT-Bio-C Temp^®^. The control group was a culture medium. Cytocompatibility was assessed by the methyltetrazolium (MTT) assay after exposure of the Saos-2 human osteoblast-like cell line to dilutions of commercial products/associations for 24 h and 72 h. The antimicrobial activity against mature *Enterococcus faecalis* biofilm was evaluated by the crystal violet assay. All commercial products/associations showed a cell viability similar to or even higher than the control group (*p* > 0.05) for both periods evaluated. *C. articulatus* essential oil associated or not with calcium hydroxide showed better antibiofilm capacity. *C. articulatus* associated or not with calcium hydroxide showed superior cytocompatibility and antimicrobial capacity, representing a promissory intracanal medicament.

## 1. Introduction

The use of intracanal medication between sessions can help clean and disinfect root canals [1]. An ideal intracanal medication should eliminate microorganisms that can survive the chemical–mechanical preparation, act as a physical–chemical barrier, and stimulate repair by mineralized tissue [2,3]. Calcium hydroxide (CH) is an excellent therapeutic option when the clinical situation requires the use of a dressing between sessions. The chemical dynamics of calcium hydroxide, demonstrated by ionic dissociation, characterize its properties [4]. The activation of tissue enzymes, such as alkaline phosphatase, which favors mineralization and the inhibitory effect on bacterial enzymes (antimicrobial property), illustrate the biological qualities of the hydroxyl and calcium ions of this medication. Although alkalinization occurs, these pH levels may be insufficient to eliminate some resistant microorganisms, notably *E. faecalis* and *Candida albicans* [5,6,7,8].

Several herbal compounds have been associated with CH in order to enhance its antimicrobial capacity [9,10]. Products, especially essential oils, are known for their antimicrobial activity [11,12]. *Cyperus articulatus* L. (*C. articulatus*), from the *Cyperaceae* family, popularly known as priprioca, has demonstrated antimicrobial, anti-inflammatory, antifungal, analgesic, antioxidant, and healing properties [13,14]. It has also been shown to exhibit potent fungicidal action against oral microorganisms [12]. Previous studies screened several essential oils and their bioactive fractions, showing a promising antimicrobial activity of *C. articulatus* essential oil against *Streptococcus mutans* and *Candida* spp. Furthermore, these studies have demonstrated by scanning electron microscopy analysis that the EOs disrupt biofilm integrity, but little is known about the effect of these EOs on the viability of oral biofilms [12]. Bersan et al. [13] showed that the crude oil from *C. articulatus* exhibited the best results of antimicrobial activity and an ability to control biofilm formation, probably due to the presence of terpenes and monoterpenes such as a-pinene, b-pinene, and *E*-pinocarveol. To date, no study has reported the antimicrobial activity of *C. articulatus* against *E. faecalis*. In addition, there is no study on the use of this essential oil in endodontics.

Bio-C Temp^®^ represents a recently launched bioceramic paste for intracanal use based on calcium silicate, featuring calcium tungstate as a radiopacifier [15]. According to the manufacturer, it has high alkalinity (pH = 12 ± 1) and may represent a new alternative in regenerative endodontic procedures. Recent studies have shown the superior ability of Bio-C Temp^®^ to induce the activity of the alkaline phosphatase enzyme compared to other intracanal medications. However, it showed lower antimicrobial activity against *E. faecalis* [16], and a recent study showed that Bio-C Temp^®^ can cause a certain degree of coronary discoloration in the long term [17].

The objective of the present study was to evaluate the cytocompatibility in a human osteosarcoma cell line Saos-2 and the antimicrobial activity of *C. articulatus* essential oil mixed or not with CH compared to the new bioceramic intracanal medicament Bio-C Temp^®^ against the mature biofilm of *E. faecalis*. The null hypothesis was that there is no difference in cytocompatibility and antibiofilm activity for the *C. articulatus* essential oil mixed or not with CH and Bio-C Temp^®^.

## 2. Results

### 2.1. Cell Viability

**Figure 1:**
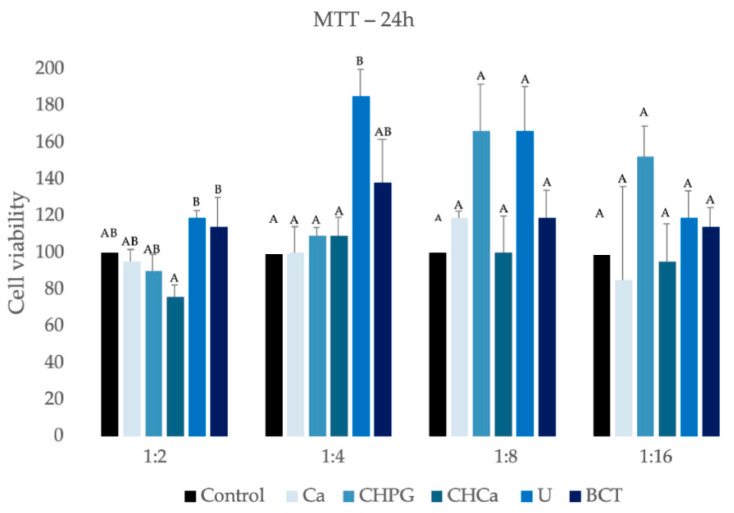
Cell viability evaluated by MTT after 24 h of exposure of Saos-2 to intracanal medicaments and to the control (culture medium). Different letters in columns indicate a significant difference among the groups in each dilution. Group Ca–*C. articulatus* essential oil; group CHPG-calcium hydroxide associated with propylene glycol; group CHCa-essential oil of *C. articulatus* associated with calcium hydroxide; group U-UltraCal^®^ XS; group BCT-Bio-C Temp^®^. Control group was culture medium.

**Figure 2:**
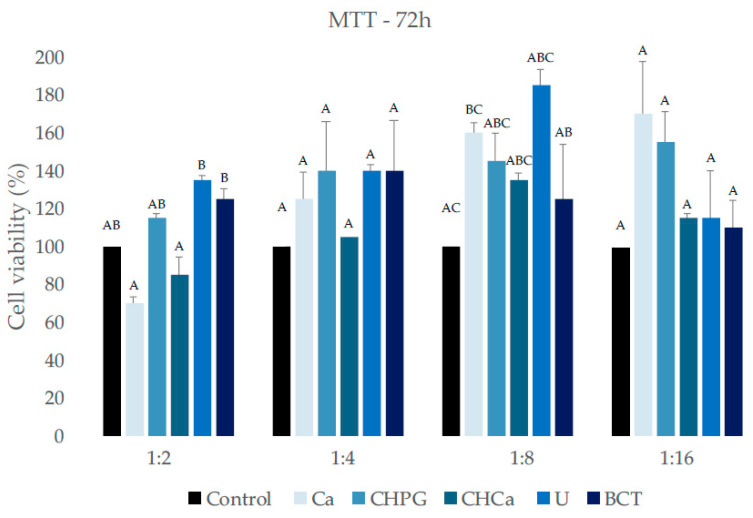
Cell viability evaluated by MTT after 72 h of exposure of Saos-2 to intracanal medicaments and to the control (culture medium). Different letters in columns indicate a significant difference among the groups in each dilution. Group Ca–*C. articulatus* essential oil; group CHPG-calcium hydroxide associated with propylene glycol; group CHCa-essential oil of *C. articulatus* associated with calcium hydroxide; group U-UltraCal^®^ XS; group BCT-Bio-C Temp^®^. Control group was culture medium.

The results of the MTT assay showed that, for 24 h and 72 h, all commercial products/associations showed cell viability similar to or even higher than the control group (*p* > 0.05) (Figure 1 and Figure 2). At the 1:2 dilution, for a period of 24 h, the U and BCT groups showed higher viability than the CHCa group. However, with no statistical difference compared to the Ca, CHPG, and control groups (Figure 1) for the 72 h period, the U and BCT groups also showed higher viability than the Ca and CHCa groups at the same dilution (Figure 2).

At the 1:4 dilution, for a period of 24 h, the U group showed higher viability than the Ca, CHCP, CHCa, and control groups, not differing statistically only from the BCT group (Figure 1).

For comparisons between the two periods of analysis (intragroup comparisons), there was no significant difference in the mean cell-viability values of the experimental and control groups (*p* < 0.05), with the exception of the Ca group, which showed a significant drop in viability only at the 1:2 dilution (Table 1).

### 2.2. Assessment of Biofilm Biomass

**Figure 3:**
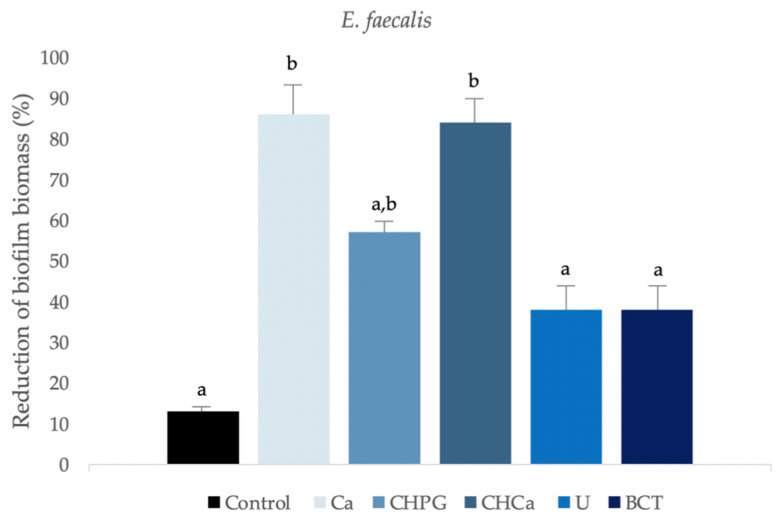
Reduction in biofilm biomass of *E. faecalis* (%) after 48 h of contact with intracanal medicaments and control. Different letters in columns indicate a significant difference among the groups. *p* < 0.001, standard deviation (SD) = 0.87. Group Ca–*C. articulatus* essential oil; group CHPG-calcium hydroxide associated with propylene glycol; group CHCa-essential oil of *C. articulatus* associated with calcium hydroxide; group U-UltraCal^®^ XS; group BCT-Bio-C Temp^®^. Control group was culture medium.

Regarding the reduction in *E. faecalis* biofilm biomass, only the Ca and CHCa groups reduced significantly compared to the control group (*p* < 0.001) (Figure 3). Groups U and BCT showed a significantly smaller percentage reduction (*p* < 0.001), not differing from the control group. Group CHPG showed a significant reduction, however, without significantly differing from the other groups (Figure 3).

## 3. Discussion

Microorganisms are the main etiological factor for the development of pulp and periradicular diseases [2]. The chemical–mechanical preparation promotes a significant reduction in the microbial load. However, some species are able to survive due to the complex anatomy of the root-canal system, as well as microbial resistance factors [1,18]. Intracanal medications can be used to complement disinfection [18,19], with CH being the most used medication due to its biological and antimicrobial activity [20], as well as its ability to inactivate bacterial endotoxin [19].

Despite its excellent properties, CH is not effective against all microorganisms isolated from infected root canals [21]. According to the study by van der Waal et al., CH favored the growth of *E. faecalis* in a two-species biofilm model [22].

The vehicle plays a critical role in the biological effectiveness of CH pastes, which is determined by the rate of ionic dissociation into Ca^2+^ and OH^−^ ions. According to Leonardo et al. [23], pastes prepared with distilled water or another water-soluble aqueous vehicle do not have good physicochemical properties, being soluble and possibly permeable to periapical tissue fluids.

Natural products, especially essential oils, have been used in the development of medicines for various therapeutic applications. The study by Freires et al. [12] demonstrated a significant reduction in the biovolume of polysaccharides in the *Streptococcus mutans* biofilm after treatment with essential oils from A. gratissima and L. medoides, while the essential oil from C. sativum showed significant action against *C. albicans*.

There is growing interest in research on the essential oil of *C. articulatus* due to its antifungal [24], antibacterial [25,26,27], and antioxidant [27] properties. In this context, the objective of the present study was to evaluate the antimicrobial capacity and cytotoxicity of CH associated with *C. articulatus* essential oil. This is the first study in the literature to evaluate the association of this essential oil with CH. Commercially available intracanal medications were also tested.

Recently, the incorporation of bioceramic compounds has brought new perspectives to endodontics [28,29]. The Bio-C Temp^®^ intracanal bioceramic paste showed alkaline pH, high calcium release, and dose-dependent cytotoxicity. However, it showed no penetration into dentinal tubules [15].

Considering the antimicrobial capacity, the null hypothesis of the present study was rejected, since there were differences between the commercial products/associations tested. This represents the first study to evaluate the antimicrobial capacity of *C. articulatus* essential oil against *E. faecalis*. Previous studies evaluated the antimicrobial effect against some oral pathogens: *C. albicans*, *Fusobacterium nucleatum*, *Porphyromonas gingivalis*, *Streptococcus sanguis*, *Streptococcus mitis*, and *Streptococcus mutans* [12,13].

In the literature, there are studies testing the antimicrobial activity of essential oils or CH associated with essential oils by determining the minimum inhibitory concentration (MIC) and the minimum bactericidal/microbicidal concentration (MBC/MMC) [11,13]. Silva et al. [30] evaluated the antimicrobial activity of different concentrations of diluted essential oils of *Rosmarinus officinalis* L., *Zingiber officinale*, *Citrus aurantium bergamia*, and *Copaifera officinalis*, alone and associated with CH against *E. faecalis*, using the broth microdilution test. Laboratory MIC tests represent a primary measurement being performed on planktonic cells, and the use of medications on their MIC does not cause any effect on microorganisms in biofilm [31].

Cosan et al. [9] evaluated the antimicrobial capacity of CH associated with the essential oils of Origanum dubium and Mentha spicata, using the agar diffusion test. Krüger et al. [10] found the antimicrobial activity of *Myracrodruon urundeuva* extract to be lower than the vancomycin control using the disk diffusion test. According to the editorial published in December 2007 in the *Journal of Endodontics*, for the agar diffusion method, there is no standardization of the medium or materials tested [32]. Chemical interactions between the media and the antimicrobial agents are largely unknown. The antimicrobial capacity of some endodontic medications is related to pH; therefore, the buffering effect of agar may determine the diameter of the growth-inhibition zone, not the actual antimicrobial efficacy. Therefore, the information obtained in studies that used the agar diffusion test does not reflect antimicrobial activity in vitro or in vivo; therefore, such a method should not be used to compare and select antimicrobial agents for clinical use.

The direct contact test was also used in several studies, as it represents a reproducible, low-cost, and easy-to-perform method [33,34]. However, it provides data on the antimicrobial capacity only in planktonic microorganisms, not simulating an in vivo situation, since endodontic infection is mainly found in the form of biofilm. Therefore, in the present study, the crystal violet test was performed to evaluate the biomass of the mature *E. faecalis* biofilm.

Guerreiro et al. [16] evaluated the antimicrobial activity of Ultracal^®^ XS (Ultradent Products, South Jordan, UT, USA) and Bio-C Temp^®^ (Angelus, Londrina, PR, Brazil) against the 24 h biofilm of *E. faecalis*. In the present study, mature biofilm grown for 7 days was used, since a more aged biofilm is recommended to evaluate the antimicrobial activity of endodontic materials [35,36].

According to the results of the crystal violet test, the essential oil of *C. articulates*, as well as its association with CH, presented an antibiofilm effect that was statistically superior to the intracanal medicaments Ultracal^®^ XS and Bio-C Temp^®^ (*p* < 0.001), which did not differ from the group control. It has been demonstrated that the main effects exerted by compounds present in essential oils are related to changes in the cell membrane and its functions [13,14,15,16,17,18,19,20,21,22,23,24,25,26,27,28,29,30,31,32,33,34,35,36,37]. The bioactive compound β-pinene can destroy cellular integrity and inhibit mitochondrial respiration and ion-transport processes [38,39]. According to the manufacturer, the *C. articulatus* essential oil tested in the present study has 6% β-pinene. Mustakone, the main compound of the essential oil from *C. articulatus* (9.8–14.5%), may also be related to the antimicrobial activity observed in this study. According to Taheri et al. [40], the wide array of biological activities is closely linked to the presence of phytochemicals, such as α-cyperone, α-pinene, caryophyllene oxide, and mustakone, suggesting that the antimicrobial activity was due to the presence of these chemical compounds. Furthermore, previous research showed strong antimicrobial activity in the essential oil from *C. articulatus* against the oral pathogens *Candida albicans*, *Fusobacterium nucleatum*, *Porphyromonas gingivalis*, *Streptococcus sanguis*, and *Streptococcus mitis*, and chemical analysis of the oil revealed the presence of the sesquiterpene mustakone as the main component [13]. The commercial oil from Quinari (Ponta Grossa, PR, Brazil) presents 9.8–14.5% mustakone, suggesting the antimicrobial property was also due to the presence of these compounds. 

Swain et al. [41] showed that compounds like mustakone could effectively bind to the receptors of the target enzyme TyrRS of *Staphylococcus aureus* with high stability and integrity. According to the authors, pharmacokinetic, drug-like properties and toxicity analysis of the EO metabolites supported the candidature of mustakone and khusinol as pharmacologically important antibacterial drug ingredients. However, the ability of *C. articulatus* essential-oil components against *E. faecalis* still needs to be explored.

Essential oils can also cross the cell membrane of fungi, interacting with membrane enzymes and proteins, thus producing a flow of protons to the outside of the cells, which induces cell death [42].

In the study by Guerreiro et al. [16], the commercial pastes Calen^®^ and UltraCal^®^ XS showed a higher capacity to reduce *E. faecalis* biofilm biomass than Bio-C Temp^®^. In the present study, the bioceramic medicine Bio-C Temp^®^ and UltraCal^®^ XS presented the lowest averages of biofilm reduction, not statistically different from the control group. This difference can be attributed to the more structured biofilm. The resistance mechanism of older biofilm is complex and may involve changes in the penetration of antimicrobial agents through the cell envelope, the production of enzymes, and the increase in the exopolysaccharide matrix during biofilm development [43,44].

Even though different types of vehicles demonstrate different chemical characteristics of dissociation and diffusibility, which are decisive in the evaluation of biological behavior [4], and pastes have different additives and different proportions of CH, the CH-propylene glycol association has a higher final percentage of CH than the commercial paste Ultracal^®^ XS.

The use of in vitro tests to analyze cell viability represents the first step in studying the biological compatibility of a substance and can provide important data related to the biocompatibility of different materials [45].

The Saos-2 cell line was selected, as it is widely used to assess cytotoxicity [28,33]. The results of the MTT assay of the present study corroborate those of previous studies that reported good cytocompatibility of CH-based intracanal drugs [16,46,47,48].

All commercial products/associations showed similar cytocompatibility to the control group in the 24 h and 72 h evaluation periods. Guerreiro et al. [16] found a lower viability of Saos-2 for the Bio-C Temp^®^ group compared to Ultracal^®^ XS, at a 1:2 dilution, within 24 h. In the present study, for a period of 72 h, there was lower viability of Saos-2 for *C. articulatus* essential oil and its association with HC compared to Bio-C Temp^®^ and Ultracal^®^ XS, in a 1:2 dilution, as well as for CHCa group, for a period of 24 h, also in a 1:2 dilution. The U group also showed greater viability for a period of 24 h, at a 1:4 dilution. MTT is a tetrazolium salt that is reduced to purple formazan crystals mainly by the mitochondrial enzyme tetrazolium-succinate dehydrogenase [49]. Theoretically, the color intensity of the formazan dye is correlated with the number of viable cells. However, some chemicals or phytochemicals can alter the activity of the succinate dehydrogenase enzyme or interact directly with MTT [50], which could justify this greater cytotoxicity for groups containing *C. articulatus* essential oil, in the dilution 1:2. However, this difference between the groups was not observed for the other dilutions, in both periods of analysis.

It is important to highlight that, for the MTT assay, a direct correlation between cell number and tetrazolium salt reduction may not occur; therefore, the simultaneous evaluation of different cellular parameters using other tests (crystal violet, neutral red, and Trypan blue) is necessary to provide reliable information on the cytotoxicity of substances/associations.

One limitation of the present study was the use of a mono-specie biofilm. Further research is needed to address the antimicrobial activity of calcium hydroxide and *C. articulatus* association using mixed-species biofilm models, as well as to test the antimicrobial capacity against biofilms developed on dentine discs. Another aspect concerning intracanal medicament that should be evaluated is the microhardness of root dentine when it is exposed to *C. articulates*, associated or not to calcium hydroxide.

The results of the present work revealed that *C. articulatus* oil, as well as CH associated with *C. articulatus* oil, showed cytocompatibility and an excellent antimicrobial effect. This association may represent a promising medication, especially in cases of endodontic infection, and can even be used in regenerative endodontic procedures.

## 4. Materials and Methods

**Figure 4:**
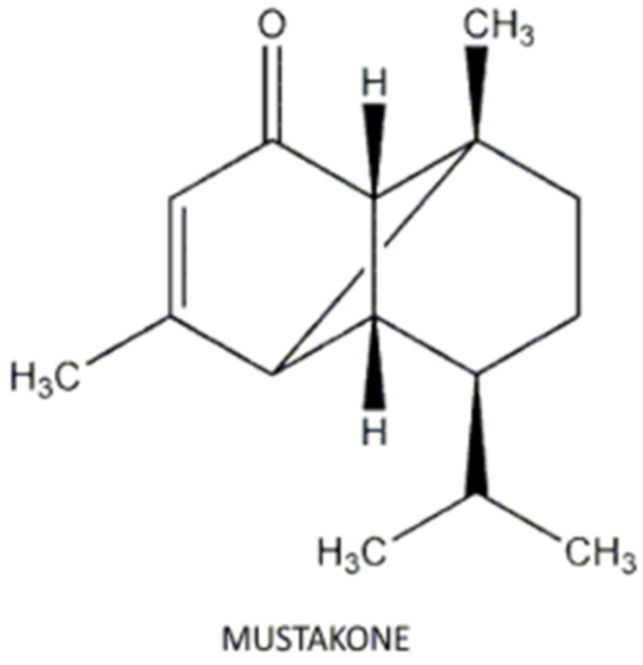
Main volatile constituent identified in the *C. articulatus* essential oil according to the manufacturer.

The experimental groups, according to the materials tested, were group 1 (Ca): *C. articulatus* essential oil; group 2 (CHPG): CH mixed to propylene glycol; group 3 (CHCa): CH mixed to propylene glycol and *C. articulatus* essential oil; group 4 (U): *UltraCal^®^ XS*; group 5 (BCT): Bio-C Temp^®^. The compositions and manufacturers of each commercial product are described in Table 2. In addition, the constituents and chemical properties of *C. articulatus* essential oil, according to the manufacturer, are presented in Table 3. Data concerning the physical properties and the complete composition of *C. articulatus* essential oil is provided in Table 3 and Figure 4.

### 4.1. Cell-Viability Assay

#### 4.1.1. Preparation of Commercial Products/Associations

A pilot study was carried out to determine the concentration of *C. articulatus* essential oil to be used in the cytocompatibility test. In this pilot study, the minimum inhibitory concentration was determined by the broth microdilution technique (unpublished data). According to the results of this pilot study, a 2 mL solution of *C. articulatus* essential oil was prepared at a concentration of 9.4 mg/mL by diluting the pure oil in propylene glycol and distilled water (group Ca).

For the CHPG group, 75 mg of CH powder were weighed on an analytical balance (Ohaus Adventurer, Model AR2140, São Bernardo do Campo, Brazil), and 75 µL propylene glycol and 3 mL distilled water were added, obtaining a suspension, for subsequent dilution in the culture medium for the Saos-2 cell line.

In the CHCa group, an aliquot of 9.4 mg/mL of *C. articulatus* essential oil was added to the paste obtained for group 2.

For the U and BCT groups, a 200 mg aliquot of each commercial product was weighed on an analytical balance and placed in Falcon tubes. Then, 2.4 mL of distilled water were added, obtaining a final concentration of 83.3 mg/mL, according to the methodology of Guerreiro et al. [16].

For all experimental groups, Dulbecco’s modified Eagle’s medium (DMEM, Sigma-Aldrich, St. Louis, MO, USA) was added and remained in an incubator at 37 °C for 24 h. Then, the supernatant was transferred to new microtubes and centrifuged at 20,800× *g* for 10 min to decant possible particles of material left in the supernatant [16]. The supernatant was transferred to new microtubes and considered the ‘mother’ solution, which was diluted (1:2, 1:4, 1:8, and 1:16) for subsequent contact with the cells.

#### 4.1.2. Cell Culture of the Saos-2 Lineage

Pre-osteoblastic cells of the Saos-2 lineage obtained from ATCC (American Type Collection, Manassas, Virginia, USA) HTB-85TM were thawed and transferred to centrifuge tubes containing 10 mL of DMEM and centrifuged at 2000 rpm for 5 min. The supernatant was discarded, and the cells were cultured in 75 cm^2^ culture flasks (Corning Incorporated, Costar, Corning, New York, NY, USA) containing DMEM supplemented with 15% fetal bovine serum (Gibco/Invitrogen Life Technologies, Grand Island, New York, NY, USA) and a 1% antibiotic–antimycotic solution (Sigma, Saint Louis, MO, USA). At subconfluence, the culture medium was removed, and 2.5 mg/mL trypsin solution (Nutricell Nutrientes Celulares, Campinas, São Paulo, Brazil) and 1 µM ethylenediaminetetraacetic acid (EDTA) (Gibco/Invitrogen Life Technologies, Grand Island, New York, NY, USA) were added to obtain cell suspension. Then, 110 cells/mm^2^ were plated in 24-well polystyrene plates (Corning Incorporated, Corning, New York, NY, USA) and cultured in McCoy’s 5A (Sigma, Saint Louis, MO, USA), an osteogenic medium consisting of DMEM supplemented with 10% fetal bovine serum (Gibco/Invitrogen Life Technologies, Grand Island, New York, NY, USA), 1% antibiotic–antimycotic solution (Sigma, St. Louis, MO, USA), 10 mM β-glycerophosphate (Sigma, Saint Louis, MO, USA), and 50 μg/mL of ascorbic acid (Gibco/Invitrogen Life Technologies, Grand Island, New York, NY, USA).

#### 4.1.3. MTT Colorimetric Assay

**Figure 5:**
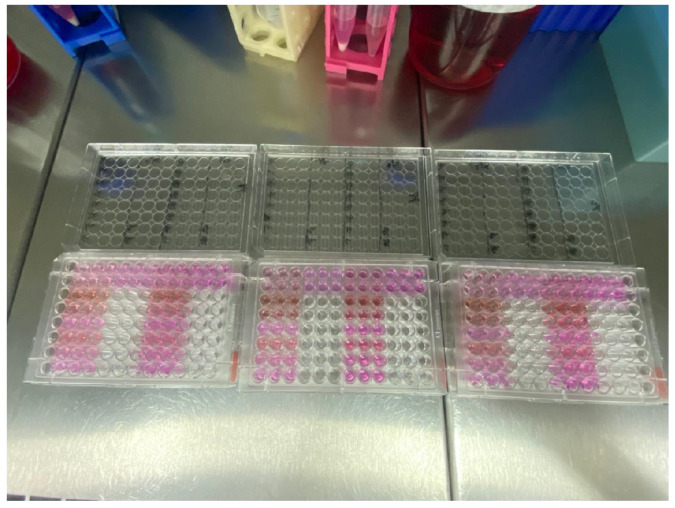
Addition of aliquots of 10 μL of the MTT to the treated cultures.

After 24 h and 72 h of plating the cells with the different commercial products/associations, aliquots of 10 μL of the MTT solution (5 mg/mL), diluted in serum-free DMEM culture medium, were added to the treated cultures (Figure 5), and these were incubated for a period of 3 h, at 37 °C, in a humid atmosphere containing 5% CO_2_ and 95% atmospheric air. After this period, the cultures were washed with 1 mL of warmed PBS (phosphate–saline) buffer solution. Then, 1 mL of 1% DMSO solution was added to each well, under stirring, for 5 min, for complete solubilization of the precipitate formed. After solubilization of the crystals, quantification was performed on an ELX800 microplate reader (Epoch, Bio-Tek Instruments, Winooski, VT, USA) at 590 nm, obtaining optical density (OD) measurements. Three independent experiments were performed in triplicate for each experimental group, and the mean of each experiment was used in the statistical analysis (*n* = 3 per group).

### 4.2. Assessment of Antimicrobial Capacity

The standard strain of *E. faecalis* (ATCC 29212) was used, which was grown overnight in Brain Heart Infusion broth (BHI, Difco, Detroit, MI, USA), plated on BHI agar (Difco, Detroit, MI, USA) and incubated at 37 °C for 24 h. The microbial suspension was prepared and adjusted to an optical density equivalent to 1 × 10^8^ CFU mL^−1^.

To carry out the microbiological tests, the preparation of the solutions/suspensions for the Ca and CHCa groups was the same as that used for the cytotoxicity tests. For the CHPG, U, and BCT groups, suspensions were prepared at a concentration of 25 mg mL^−1^ according to the methodology of de Araújo-Lopes et al. [34] and Guerreiro et al. [16].

#### Quantification of Biofilm Formation

The activity of commercial products/associations on the biomass of the mature *E. faecalis* biofilm was tested by the crystal violet assay.

**Figure 6:**
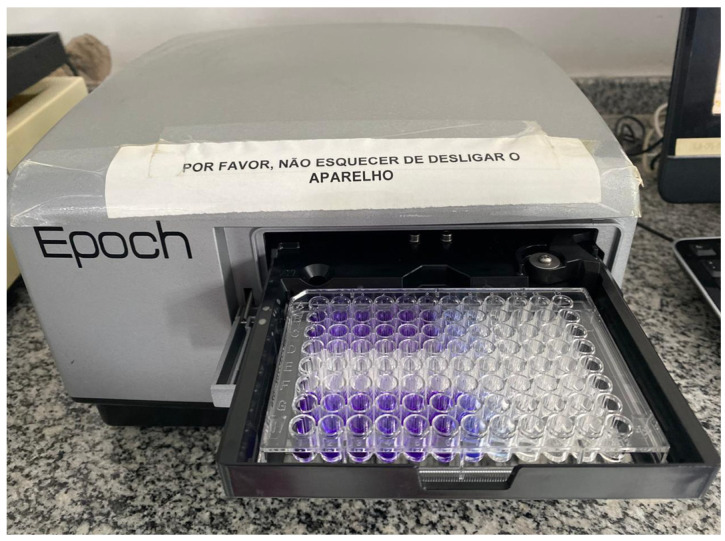
Optical density (OD) measurements in spectrophotometer.

The *E. faecalis* biofilm was formed in 96-well culture plates (NEST Biotechnology, Wuxi, China) with BHI culture medium and stored in an incubator at 37 °C for 7 days. After incubation, the wells were washed 3 times with 200 μL of sterile saline. Then, 200 μL of the suspension of each association/commercial product were added to the wells, remaining in contact with the biofilm for 48 h, in an incubator at 37 °C. A culture medium with inoculum was used as a positive control, and a well with sterile saline solution without biofilm was used as a negative control. Then, the wells were washed and fixed with methanol, and the biofilm was, then, stained with 1% crystal violet dye (Labsynth, Diadema, Brazil) for 10 min. Excess dye was washed off with a thorough rinse. Then, 200 μL of 33% acetic acid (Merck, Boston, MA, USA) were added to elute the biofilm, and 50 μL were transferred to Elisa microplates and measured in a spectrophotometer (Epoch, Biotek, Winooski, VT, USA) at a wavelength of 590 nm (Figure 6). The intensity of biofilm formation by the bacterial strain was correlated with the incorporation of crystal violet. The results were presented as a percentage reduction in biofilm biomass (%).

All the experiments were performed by the same operator, and all tests were conducted in triplicate according to the dilution/group for cytocompatibility tests and according to the group for antibiofilm activity tests.

### 4.3. Statistical Analysis

The data obtained from the experiments were analyzed using IBM SPSS statistical software (version 26.0, IBM Corporation, Armonk, New York, NY, USA). The cell-viability values obtained were compared between the experimental groups using the one-way ANOVA test, with Tukey’s multiple comparisons post-test, and then, for each of the groups, the viability values were compared over time of prospecting (24 h and 72 h) using the paired Student’s *t* test. The percentage of cell viability was also evaluated by comparing the groups using Pearson’s chi-square test with Bonferroni adjustment and comparing the percentages over the prospecting time using Cochran’s Q test. The distribution graphs of cell-viability percentages were constructed using the statistical software GraphPad Prism (version 9.0.0, GraphPad Prism, GraphPad Software, San Diego, CA, USA). For all tests, a significance level of 5% was adopted.

For biomass analysis, data were analyzed using the one-way ANOVA test with Tukey HSD multiple comparisons post-test after the bootstrapping resampling process to correct deviations from sample normality. The effect size (Cohen’s f), as well as the power of the study, were calculated using the statistical software G*Power (version 3.1.9.4, Heinrich-Heine, Universität Düsseldorf, Düsseldorf, Germany). For all tests, a significance level of 5% was adopted.

## 5. Conclusions

All tested drugs showed no cytotoxic effects within 24 h and 72 h. *C. articulatus* oil showed an excellent antimicrobial effect, and its association with CH also showed a high capacity to reduce mature *E. faecalis* biofilm. The Bio-C Temp^®^ medication had a lower antimicrobial and antibiofilm effect.

## Figures and Tables

**Table 1:** Mean and standard deviation (SD) values of optical density for Saos-2 cell viability evaluated by MTT and comparison between experimental and control groups at different study evaluation times (24 and 72 h, *n* = 3).

Dilution	Group	24 h	72 h	*p*
1:2	Control	0.21 (0.03) AB,a	0.20 (0.03) AB,a	0.715
	Ca	0.20 (0.02) AB,a	0.14 (0.02) A,b	0.013
	CHPG	0.19 (0.02) AB,a	0.23 (0.01) AB,a	0.238
	CHCa	0.16 (0.01) A,a	0.17 (0.03) A,a	0.614
	U	0.25 (0.01) B,a	0.27 (0.01) B,a	0.524
	BCT	0.24 (0.03) B,a	0.25 (0.02) B,a	0.763
	*p*	0.008	0.001	
1:4	Control	0.21 (0.03) A,a	0.20 (0.03) A,a	0.715
	Ca	0.21 (0.03) A,a	0.25 (0.03) A,a	0.174
	CHPG	0.23 (0.01) A,a	0.28 (0.11) A,a	0.593
	CHCa	0.23 (0.03) A,a	0.21 (0.00) A,a	0.464
	U	0.39 (0.07) B,a	0.28 (0.01) A,a	0.100
	BCT	0.29 (0.06) AB,a	0.28 (0.11) A,a	0.948
	*p*	0.005	0.513	
1:8	Control	0.21 (0.03) A,a	0.20 (0.03) AC,a	0.715
	Ca	0.25 (0.01) A,a	0.32 (0.05) BC,a	0.175
	CHCP	0.35 (0.09) A,a	0.29 (0.03) ABC,a	0.364
	CHCa	0.21 (0.04) A,a	0.27 (0.01) ABC,a	0.211
	U	0.35 (0.08) A,a	0.37 (0.05) ABC,a	0.772
	BCT	0.25 (0.03) A,a	0.25 (0.06) AB,a	0.943
	*p*	0.056	0.006	
1:16	Control	0.21 (0.03) A,a	0.20 (0.03) A,a	0.715
	Ca	0.18 (0.11) A,a	0.34 (0.09) A,a	0.993
	CHCP	0.32 (0.03) A,a	0.31 (0.04) A,a	0.959
	CHCa	0.20 (0.03) A,a	0.23 (0.01) A,a	0.123
	U	0.25 (0.02) A,a	0.23 (0.06) A,a	0.711
	BCT	0.24 (0.02) A,a	0.22 (0.03) A,a	0.570
	*p*	0.128	0.059	

Group Ca–*C. articulatus* essential oil; group CHPG-calcium hydroxide associated with propylene glycol; group CHCa-essential oil of *C. articulatus* associated with calcium hydroxide; group U-UltraCal^®^ XS; group BCT-Bio-C Temp^®^. Control group was culture medium. One-way ANOVA test, with Tukey’s multiple comparison post-test (Comparison in the column. Different capital letters indicate a statistically significant difference between groups). Paired Student’s *t*-test (Comparison on the line. Different lowercase letters indicate a statistically significant difference between groups). Significance level = 5%.

**Table 2:** Tested materials, composition, and manufacturers.

Material	Manufacturer	Composition
calcium hydroxide	Biodinamica Química e Farmacêutica LTDA., Paraná, Brazil	calcium hydroxide (10 g)
*C. articulatus* essential oil	Quinari^®^, Ponta Grossa, PR, Brazil	*C. articulatus* pure oil (10 mL)
*UltraCal^®^ XS*	Ultradent Products Inc., South Jordan, UT, USA	calcium hydroxide, barium sulfate, and methylcellulose, in aqueous solution.
Bio-C Temp^®^	Angelus Indústria de Produtos Odontológicos S/A, Londrina, PR, Brazil	Calcium silicates, calcium aluminate, calcium oxide, calcium tungstate, and titanium oxide.

**Table 3:** Constituents and chemical properties of *C. articulatus* essential oil according to the manufacturer.

Chemical Composition/Properties	Constituents/Values
DENSITY (20 °C)	0.956
REFRACTION INDEX (20 °C)	1.5041
GAS CHROMATOGRAPHY–MASS SPECTROMETRY (GC-MS) ANALYSIS	Mustakone: 9.8–14.5% β-Caryophyllene oxide: 9% α-pinene: 5.7–12.3% β-Pinene: 6% (E)-Pinocarveol: 5% Myrtenal + myrtenol: 5%
EXTRACTION MODE	Steam distillation of tubers

## Data Availability

All data, tables, and figures in this manuscript are original. The raw data from this experiment are fully available upon request to the corresponding author.
